# Database study of risk factors for breast cancer-related lymphedema: a statistical analysis of 2359 cases over 10 years

**DOI:** 10.1007/s00595-024-02960-5

**Published:** 2024-11-19

**Authors:** Akihiro Matsumoto, Kai Ushio, Hiroaki Kimura, Shinichi Tomioka, Shinsuke Sasada, Makoto Asaeda, Yuki Nakashima, Koki Fukuhara, Yukio Mikami

**Affiliations:** 1 Collaborative Research Division of Medical Care Design in Indonesia, Hiroshima University Hospital, 1-2-3 Kasumi, Minami-ku, Hiroshima, Hiroshima Japan; 2 Department of Rehabilitation Medicine, Hiroshima University Hospital, 1-2-3 Kasumi, Minami-ku, Hiroshima, Hiroshima Japan; 3 Department of Public Health and Health Policy, Hiroshima University, 1-2-3 Kasumi, Minami-ku, Hiroshima, Hiroshima Japan; 4 Research Institute for Radiation Biology and Medicine, Hiroshima University Hospital, 1-2-3 Kasumi, Minami-ku, Hiroshima, Hiroshima Japan; 5 Division of Rehabilitation, Department of Clinical Practice and Support, Hiroshima University Hospital, 1-2-3 Kasumi, Minami-ku, Hiroshima, Hiroshima Japan

**Keywords:** Breast cancer, Lymphedema, Risk factor, Multivariate logistic regression analysis, Database study

## Abstract

**Purpose:**

Identifying risk factors for breast cancer-related lymphedema (BCRL) is crucial for its prevention, necessitating large-scale epidemiological studies. Despite their suitability for large-scale surveys, to our knowledge, databases have not been the basis of any study done to investigate BCRL risk factors. This study aimed to test the hypothesis that a database-based study would be useful for identifying BCRL risk factors.

**Methods:**

Patients with breast cancer diagnosed between April 2009 and March 2020 were identified from the Hiroshima University Hospital’s medical database. This retrospective observational study validated the risk factors for BCRL using logistic regression analysis (*p* < 0.05).

**Results:**

Among the total 4471 breast cancer patients identified, 2359 met the study criteria, with a BCRL incidence of 4.8%. Identified risk factors included obesity with a BMI of 25–30 (OR = 3.066, 95% CI 1.408–6.677), severe obesity with a BMI > 30 (OR = 5.791, 95% CI 2.239–14.97), surgical axillary lymph node dissection (OR = 3.212, 95% CI 1.918–5.378), chemotherapy with docetaxel (OR = 1.795, 95% CI 1.062–3.032), and conventional radiation to the breast or chest wall including lymph nodes in the irradiated area (OR = 3.299, 95% CI 1.842–5.910).

**Conclusions:**

The BCRL risk factors identified by our database analysis were in line with those documented in previous studies, indicating the usefulness of database-based studies. Future studies should include more patients and study items.

## Introduction

Breast cancer-related lymphedema (BCRL) is a physical and socio-psychological issue for patients with breast cancer, which reduces their activities of daily living (ADL) and quality of life substantially [1–6]. More than 2.08 million new cases of breast cancer were reported worldwide in 2018 [7] and improving survival rates [8, 9] have resulted in an increased cumulative risk of BCRL, escalating healthcare costs [10].

Although the pathogenesis of BCRL is not fully understood, it is believed to be associated with the destruction of lymphatic tissue caused by breast cancer treatment [11, 12], often occurring within the first year after surgery [6, 11, 12]. BCRL treatment includes manual therapy, elastic bandage compression therapy, and surgery [1, 13]; however, resolution is difficult once BCRL develops. Conversely, preventive interventions can reduce the severity and delay the onset of lymphedema [14]. Therefore, identifying its risk factors is crucial to determine preventive interventions for high-risk patients [1, 3].

Obesity, mastectomy, axillary lymph node dissection, chemotherapy, and radiation to axillary lymph nodes are all risk factors for BCRL [6, 15–21]. However, these risk factors differ among studies, with some reporting that obesity does not affect BCRL [6, 17] and some reporting that mastectomy is not a risk factor for BCRL [17–19]. Taxanes were identified as a risk factor by some studies [15, 18, 19], and not by others [17], with certain findings suggesting that chemotherapy is not a risk factor [21]. These discrepancies may stem from differences in participant numbers, study periods, and study items. Increasing the sample size can reduce result variability, highlighting the need for a comprehensive study of BCRL risk factors with a larger sample size and extensive survey items.

Database-based studies have gained recent popularity owing to the increased size and scope of studies. These studies are being used increasingly to investigate disease risk factors [22–25]. For breast cancer, database-studies have examined the treatment selection and epidemiology of male breast cancer [26, 27]; however, no studies have used databases to identify the risk factors for BCRL. Demonstrating the usefulness of database-based studies for identifying BCRL risk factors could improve prevention activities. The present study tested the usefulness of analyzing database-based studies of a large number of patients and survey items, to identify the risk factors for BCRL. Our findings demonstrate the usefulness of database-based studies for identifying the risk factors for BCRL. Thus, we conducted a study on the risk factors for BCRL using our expanded medical database.

## Materials and methods

### Data source

We used the medical database of Hiroshima University Hospital to collect patient age, sex, height, weight, and diagnosis according to the ICD-10. All treatment data were obtained from procedures performed at this hospital. The database conforms to the Diagnosis Procedure Combination (DPC), a national database in Japan, and an electronic receipt processing system. Physicians recorded all diagnoses in accordance with ICD-10 based on medical record entries, and medical clerks recorded all tests and treatments performed. As the results of pathological diagnoses at the time of surgery and data on treatments performed at other medical facilities were not recorded in the Hiroshima University Hospital database, this information was collected via a medical record review. Therefore, this study used a combination of data extracted from a single hospital database, similar in structure to the national database in Japan, and data obtained from a retrospective review of the medical records of that hospital.

### Study participants

The subjects of this study were patients who underwent surgery for diagnosed breast cancer at this major teaching hospital during the 10-year period from April 2009 to March 2020. Breast cancer diagnoses were extracted using the ICD-10 codes for breast cancer recorded in the medical database. For the diagnosis of BCRL, the breast surgeon and occupational therapist at our hospital measured the circumference of both upper extremities of the patient. When the difference in circumference was > 1 cm from right to left, upper extremity lymphedema was diagnosed and the relevant ICD-10 code was recorded. In this study, the ICD-10 codes for upper extremity lymphedema recorded after breast cancer surgery were used to diagnose BCRL.

Patients whose lymphedema developed prior to the start of breast cancer treatment, those who did not undergo surgical treatment at our institution, those whose height or weight data were not recorded in the database, and those whose postoperative observation ended within less than 1 year were excluded from the final analysis.

### Study design

This was a retrospective observational study that used a database and a medical record review. Patient consent for this study was waived because the patients provided written consent at the start of treatment for the use of their personal information for research purposes, as long as they were not personally identified. Formal consent was not required for this study.

### Survey items

Patient age, height, weight, comorbidities, and information related to breast cancer, including surgery, pathology, chemotherapy, and radiation therapy, were extracted from the database and medical records. Considering the evidence from previous studies that young age and age > 65 are risk factors for breast cancer, we divided the patients into three age groups: ≤ 34 years, 35–64 years, and ≥ 65 years. BMI calculated from height and weight was classified into four groups: underweight (< 18.5), normal weight (18.5–25), obese (25–30), and severely obese (> 30), according to the BMI distribution among the Japanese population. Comorbidities included hypertension, diabetes mellitus, and hyperlipidemia being treated as metabolic diseases. Diagnoses were extracted using ICD-10 codes and prescription records of medications for these diseases. Information related to breast cancer treatment included surgery: bilateral mastectomy, total mastectomy, partial mastectomy, sentinel lymph node biopsy, axillary lymph node dissection, and breast reconstruction; pathology: non-invasive and invasive cancer; chemotherapy: all anticancer drugs used; radiation therapy: whether the irradiation method was conventional or hypofractionated, whether lymph nodes were included in the irradiated area, and whether boost irradiation to tumor nests was performed.

### Statistical analyses

The chi-square test evaluated the differences in clinical characteristics between BCRL-affected and non-affected patients. Logistic regression analysis investigated the risk factors for BCRL, with age, BMI, comorbidities, surgery, pathology, chemotherapy, and irradiation, defined as independent variables. The significance level was set at *p* < 0.05. Stata MP version 15.1 (StataCorp LP, College Station, TX, USA) was used for statistical analysis.

## Results

**Fig. 1:**
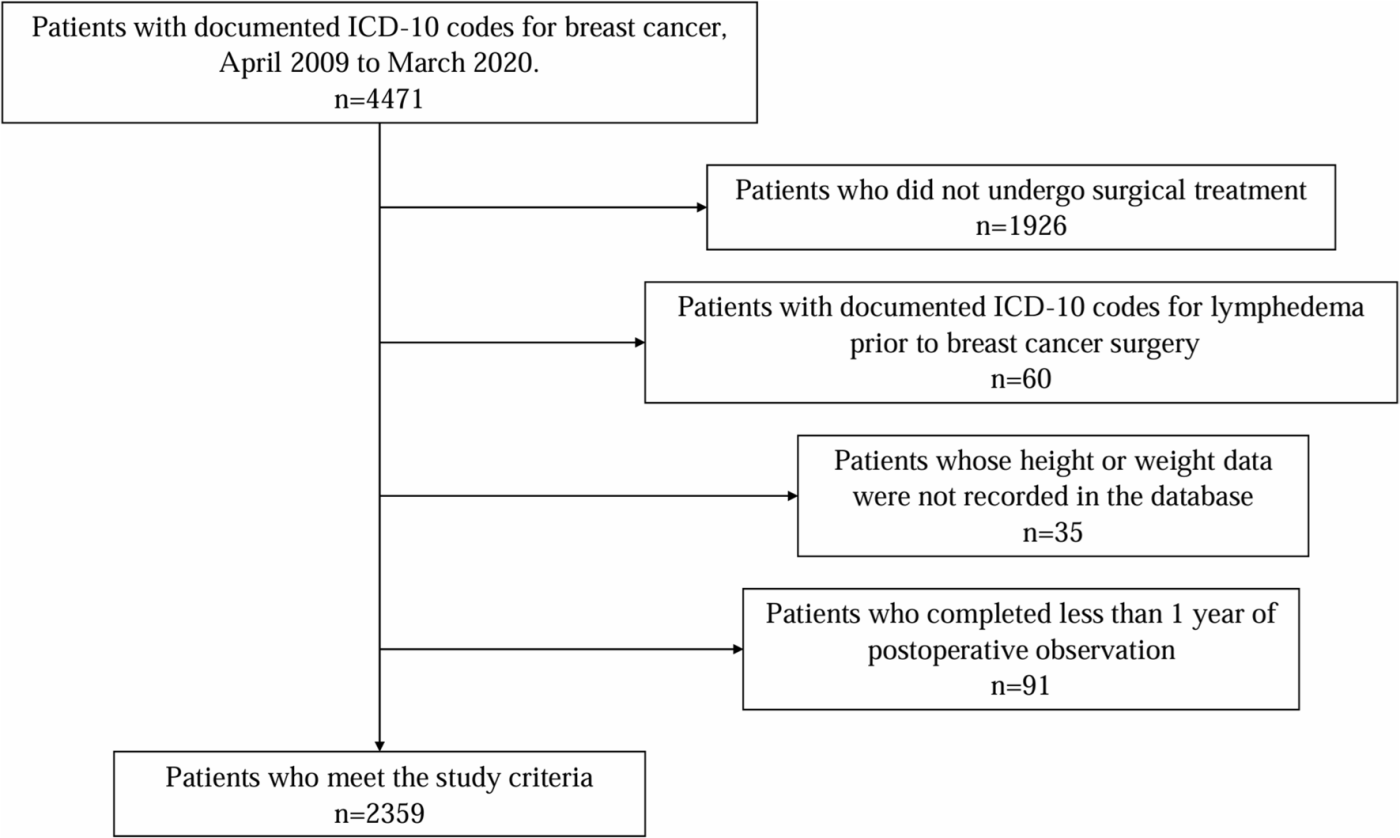
XXXX

Among a total of 4471 patients with breast cancer in our hospital database, 2359 fulfilled the study criteria and were the subjects of this analysis. Figure 1 shows the flowchart of the data collection process. BCRL was diagnosed in 114 patients, with an incidence of 4.8%. Table 1 summarizes the patients’ characteristics. The mean age of the patients was 57.8 ± 13.4 years. According to the BMI results, 18.3% of the patients were obese and 4.2% were severely obese. The comorbidities during treatment included hypertension (5.6%), diabetes mellitus (2.8%), and hyperlipidemia (5.7%). Regarding surgical treatment, total mastectomy and axillary lymph node dissection were performed in 47.6% and 43.8% of patients, respectively. Postoperative pathology revealed invasive cancer in 65.3%. Chemotherapy was administered to 26.8% of the patients, including the anthracycline epirubicin, taxanes, docetaxel and paclitaxel, and the anti-HER2 agents, trastuzumab and pertuzumab. Radiotherapy was given to 60.0% of the patients. Radiotherapy that did not include lymph nodes in the irradiated area consisted of both conventional and hypofractionated irradiation, whereas radiotherapy that included lymph nodes in the irradiated area consisted of conventional irradiation alone.

**Table 1:** Summary of factors for the breast cancer patents

Patient factors	Subgroup	Total patients		BCRL(−)		BCRL(+)	
		Number	Incicence (%)	Number	Incicence (%)	Number	Incicence (%)
whole patients	–	2359	–	2245	95.2	114	4.8
Age	< 36 years old	71	3.0	67	94.4	4	5.6
	36 ~ 64 years old	1475	62.5	1401	95.0	74	5.0
	> 64 years old	813	34.5	777	95.6	36	4.4
BMI	< 18.5 kg/cm2	254	10.8	244	96.1	10	3.9
	18.5 kg/cm2 ~ 25 kg/cm2	1575	66.8	1518	96.4	57	3.6
	25 kg/cm2 ~ 30 kg/cm2	433	18.4	399	92.1	34	7.9
	> 30 kg/cm2	97	4.1	84	86.6	13	13.4
Hypertension	ICD-10 code with treatment drug	132	5.6	119	90.2	13	9.8
Diabitase	ICD-10 code with treatment drug	67	2.8	62	92.5	5	7.5
Hyperlipidemia	ICD-10 code with treatment drug	135	5.7	125	92.6	10	7.4
Surgery	Bilateral surgery	134	5.7	127	94.8	7	5.2
	Total mastectomy	1124	47.6	1051	93.5	73	6.5
	Partial mastectomy	1235	52.4	1194	96.7	41	3.3
	Sentinel lymph node biopsy	1325	56.2	1303	98.3	22	1.7
	Axillary lymph node dissection	1034	43.8	942	91.1	92	8.9
	Breast reconstruction	218	9.2	210	96.3	8	3.7
Pathology	Non-invasive cancer	819	34.7	790	96.5	29	3.5
	Invasive cancer	1540	65.3	1455	94.5	85	5.5
Chemotherapy	Epirubicin—Anthracycline	479	20.3	423	88.3	56	11.7
	Docetaxel—Taxane	677	28.7	613	90.5	64	9.5
	Paclitaxel—Taxane	193	8.2	171	88.6	22	11.4
	Trastuzumab—Anti-HER2 humanized monoclonal antibody	231	9.8	206	89.2	25	10.8
	Pertuzumab—Anti-HER2 humanized monoclonal antibody	49	2.1	44	89.8	5	10.2
Radiationtherapy	Normal radiotherapy to breast/chest wall	769	32.6	735	95.6	34	4.4
	Hypofractionated radiotherapy to breast/chest wall	383	16.2	377	98.4	6	1.6
	Conventional radiotherapy to lymph nodes	264	11.2	216	81.8	48	18.2
	Hypofractionated radiotherapy to lymph nodes	0	0.0	0	0.0	0	0.0
	Boost irradiation to tumor nests	495	21.0	472	95.4	23	4.6

Table 2 presents the results of the univariate logistic regression analysis. The significant independent variables were a BMI of 18.5– < 25, a BMI of 25– < 30, a BMI of ≥ 30, treated hypertension, total mastectomy, manual axillary lymph node dissection, pathologic diagnosis of invasive cancer, chemotherapy with epirubicin, docetaxel, paclitaxel, trastuzumab, and oligofractionated radiotherapy to the breast or chest wall not including the lymph nodes, and conventional radiotherapy to the breast or chest wall including lymph nodes in the irradiated area.

**Table 2:** Results of simple logistic regression analysis of the patient factors

Patient factors	Subgroup	Odds ratio	95% CI	*p* value
Age	< 36 years old	1.182	0.423 ~ 3.300	0.749
	36 ~ 65 years old	1.114	0.751 ~ 1.652	0.590
	> 65 years old	0.871	0.581 ~ 1.306	0.507
BMI	< 18.5 kg/cm^2^	0.788	0.406 ~ 1.529	0.482
	18.5 kg/cm^2^ ~ 25 kg/cm^2^	0.478	0.328 ~ 0.698	< 0.001
	25 kg/cm^2^ ~ 30 kg/cm^2^	1.966	1.297 ~ 2.979	0.001
	> 30 kg/cm^2^	3.311	1.786 ~ 6.138	< 0.001
Hypertension	ICD-10 code with treatment drug	2.299	1.254 ~ 4.216	0.007
Diabitase	ICD-10 code with treatment drug	1.615	0.636 ~ 4.098	0.313
Hyperlipidemia	ICD-10 code with treatment drug	1.630	0.831 ~ 3.198	0.155
Surgery	Bilateral surgery	1.091	0.497 ~ 2.392	0.828
	Total mastectomy	2.022	1.367 ~ 2.991	< 0.001
	Axillary lymph node dissection	5.784	3.605 ~ 9.279	< 0.001
	Breast reconstruction	0.731	0.351 ~ 1.521	0.403
Pathology	Invasive cancer	1.591	1.034 ~ 2.447	0.034
Chemotherapy	Epirubicin—Anthracycline	4.158	2.838 ~ 6.094	< 0.001
	Docetaxel—Taxane	3.407	2.327 ~ 4.990	< 0.001
	Paclitaxel—Taxane	2.900	1.775 ~ 4.737	< 0.001
	Trastuzumab—Anti-HER2 humanized monoclonal antibody	2.780	1.744 ~ 4.431	< 0.001
	Pertuzumab—Anti-HER2 humanized monoclonal antibody	2.294	0.892 ~ 5.902	0.085
Radiationtherapy	Normal radiotherapy to breast/chest wall	0.873	0.578 ~ 1.316	0.517
	Hypofractionated radiotherapy to breast/chest wall	0.275	0.120 ~ 0.630	0.002
	Conventional radiotherapy to lymph nodes	6.831	4.591 ~ 10.16	< 0.001
	Boost irradiation	0.949	0.594 ~ 1.516	0.828

Table 3 shows the results of the multivariate logistic regression analysis. The significant independent variables were a BMI of between 25 and 30, a BMI of > 30, axillary lymph node dissection for surgery, docetaxel for chemotherapy, and conventional radiation therapy to the breast or chest wall including the lymph nodes in the irradiated area.

**Table 3:** Results of multiple logistic regression analysis of the patient factors

Patient factors	Subgroup	Odds ratio	95% CI	*p* value
Age	> 65 years old	1.185	0.743 ~ 1.889	0.474
BMI	18.5 kg/cm2 ~ 25 kg/cm2	1.193	0.578 ~ 2.462	0.633
	25 kg/cm2 ~ 30 kg/cm2	3.066	1.408 ~ 6.677	0.005
	> 30 kg/cm2	5.791	2.239 ~ 14.97	< 0.001
Hypertension	ICD-10 code with treatment drug	1.616	0.758 ~ 3.442	0.213
Diabitase	ICD-10 code with treatment drug	0.801	0.277 ~ 2.316	0.683
Hyperlipidemia	ICD-10 code with treatment drug	1.100	0.494 ~ 2.446	0.815
Surgery	Bilateral surgery	1.203	0.518 ~ 2.794	0.666
	Total mastectomy	1.399	0.784 ~ 2.496	0.255
	Axillary lymph node dissection	3.212	1.918 ~ 5.378	< 0.001
Pathology	Invasive cancer	1.235	0.771 ~ 1.978	0.379
Chemotherapy	Epirubicin—Anthracycline	1.355	0.791 ~ 2.320	0.268
	Docetaxel—Taxane	1.795	1.062 ~ 3.032	0.029
	Paclitaxel—Taxane	1.796	0.981 ~ 3.286	0.057
	Trastuzumab—Anti-HER2 humanized monoclonal antibody	1.724	0.964 ~ 3.084	0.066
	Pertuzumab—Anti-HER2 humanized monoclonal antibody	0.682	0.227 ~ 2.041	0.494
Radiotherapy	Conventional radiotherapy to breast/chest wall	1.739	0.886 ~ 3.412	0.107
	Hypofractionated radiotherapy to breast/chest wall	0.716	0.251 ~ 2.039	0.532
	Conventional radiotherapy to lymph nodes	3.299	1.842 ~ 5.910	< 0.001
	Boost irradiation	1.106	0.632 ~ 1.935	0.722

## Discussion

This study investigated the risk factors for BCRL in 2359 patients with breast cancer, who underwent surgery at our hospital over a 10 year period. The incidence of BCRL was 4.8% and the following risk factors were identified: body mass index (BMI) > 25 and < 30, BMI > 30, surgical axillary lymph node dissection, chemotherapy with docetaxel, and conventional radiation to the breast or chest wall with lymph nodes in the irradiated area.

Table 4 summarizes the study participants, number of patients, number and frequency of BCRL, country, data source, and observation period for previous studies [6, 15–21, 28] and for this study, when details on the participants and BCRL diagnostic criteria were clearly stated. The incidence of BCRL in previous studies ranged from 5.3 to 34.0%, which is higher than in the present study. This is because the diagnosis of BCRL is based on subjective symptoms and objective findings, whereas the diagnostic criteria are not standardized [1, 5, 17]. Moreover, the incidence of BCRL may have been suppressed because our hospital provides lymphedema prevention education to patients who have undergone surgery for breast cancer, which may have influenced the results.

**Table 4:** Comparison of previous studies with the present study

Study	Number of patients	criterion	Number of BCRL (incidence)	Country	Data resource	Observation period
The present study	2359	ICD-10 code	103 (4.4%)	Japan	Medical care database & Medical record description	10 years
Zou—cited papers 7	387	objective measurement	78 (20.2%)	China	Interview	2 years
Aoishi—cited papers 17	1069	objective measurement	91 (8.5%)	Japan	Medical record description	6 years
Basta—cited papers 18	3136	ICD-9 code	325 (10.4%)	USA	Medical record description	13 years
Kaufman—cited papers 19	215	bioimpedance spectroscopy	21 (9.8%)	USA	Following prospective	6 years
Nguyen—cited papers 21	1794	medical record include words related edema	209 (11.6%)	USA	Medical record description	20 years
Khanna—cited papers 22	98	objective measurement	23 (23.5%)	India	Following prospective	2 years
Ugur—cited papers 23	455	objective measurement	124 (27.3%)	Turkey	Medical record description	25 years
Swaroop—cited papers 24	1121	perometer measurement	59 (5.3%)	USA	Medical record description	7 years
Kilgore—cited papers 34	146	bioimpedance spectroscopy	49 (34.0%)	USA	Medical record description	3 years

Aoishi et al. investigated the BCRL risk factors in Japanese patients and found that obesity did not affect BCRL [15]. Conversely, in this study of Japanese participants, obesity was found to significantly increase the risk of BCRL developing. Several obesity-related studies on BCRL prevention have been conducted. A previous study that used a weight-loss intervention after surgery reported no difference in BCRL incidence between the intervention and nonintervention groups [29]. In contrast, a study that used exercise therapy as a BCRL prevention intervention reported that the intervention group showed improvements in both the incidence and severity of BCRL [30–32]. Another study investigating the impact of exercise intervention on the life expectancy of patients with breast cancer reported improved life expectancy for the intervention group [33]. Based on the results of previous studies and the current study, patients with a higher BMI at the time of surgery are at a greater risk of BCRL, regardless of race. However, exercise therapy after breast cancer surgery may have a positive impact on the prevention of BCRL and the outcome of patients with breast cancer.

BCRL developed in 9.7% of the patients who received chemotherapy. Multivariate analysis identified that docetaxel was a significant risk factor for BCRL in this study. Docetaxel induces abnormal microvascular permeability and protein leakage caused by endothelial dysfunction [34, 35]. A study using an increase in upper extremity volume for BCRL diagnosis found that docetaxel caused mild, but not severe edema [36]. However, a study using the International Lymphedema Society criteria, including compromised patient quality of life, reported that docetaxel was an irreversible edema risk factor [15, 37].

Conventional irradiation of the breast or chest wall with lymph nodes in the irradiated area was a risk factor for BCRL. Neither conventional nor hypofractionated radiation, which did not include lymph nodes in the irradiated area, had a significant effect on BCRL. These results were in accordance with those reported in previous studies [17–19, 37]. Boost irradiation of tumor nests did not affect BCRL. Thus, in the present study, conventional irradiation to the breast or chest wall, including lymph nodes in the irradiated area, was considered a risk factor for BCRL. Thus, the risk factors for BCRL identified in this study were obesity with a BMI > 25, surgical axillary lymph node dissection, chemotherapy with docetaxel, and conventional radiation to the breast or chest wall with lymph nodes in the irradiated area. These findings were consistent with those of previous studies [6, 15–21], indicating that database-based studies are effective for identifying the risk factors for BCRL.

This study had several limitations. First, the diagnosis and treatment selection methods may have been biased by the reliance on data from a single institution, where our institution is involved in developing new treatments. Second, clinical features were not recorded in our database, and medical records were not considered. Thus, BCRL in this study may have been underestimated because factors, such as joint range of motion, extremity circumference and volume, edema characteristics, and lymphedema severity post-onset, were not considered. Moreover, cases of BCRL occurring after the follow-up period were not included. Third, it was difficult to rigorously assess the impact of patient background and treatment on BCRL development. In real-world settings, breast cancer treatment is complex, involving surgery, chemotherapy, and radiation therapy, making it difficult to rule out reciprocal influences. To overcome these limitations and identify BCRL risk factors in more detail, further studies are needed to generate data from a larger number of patients and patient factors in a multicenter setting. In this study, we combined data extracted from a single hospital database, similar in structure to the national database in Japan, with data obtained from a retrospective review of medical records from the same hospital. Combining database data with the clinical characteristics of patients recorded in medical records allows for a comprehensive big data study of BCRL risk factors.

## Conclusion

This database-based study investigated the risk factors for BCRL in 2359 patients with breast cancer, who underwent surgery at a single institution over a 10 year period. The incidence of BCRL in this study was lower than that reported in previous studies. However, the risk factors were similar to those reported in previous studies, indicating that database-based studies are useful for identifying BCRL risk factors. To further elucidate the risk factors for BCRL, a multicenter study with a larger patient population and more comprehensive study items is needed.
